# Reliable and efficient solution of genome-scale models of Metabolism and macromolecular Expression

**DOI:** 10.1038/srep40863

**Published:** 2017-01-18

**Authors:** Ding Ma, Laurence Yang, Ronan M. T. Fleming, Ines Thiele, Bernhard O. Palsson, Michael A. Saunders

**Affiliations:** 1 Stanford University, Dept of Management Science and Engineering, Stanford, CA 94305, USA; 2 University of California, San Diego, Dept of Bioengineering, La Jolla, CA 92093, USA; 3 University of Luxembourg, Luxembourg Centre for Systems Biomedicine, L-4365 Esch-sur-Alzette, Luxembourg; 4 Technical University of Denmark, Novo Nordisk Foundation Center for Biosustainability, 2970 Hørsholm, Denmark

## Abstract

Constraint-Based Reconstruction and Analysis (COBRA) is currently the only methodology that permits integrated modeling of Metabolism and macromolecular Expression (ME) at genome-scale. Linear optimization computes steady-state flux solutions to ME models, but flux values are spread over many orders of magnitude. Data values also have greatly varying magnitudes. Standard double-precision solvers may return inaccurate solutions or report that no solution exists. Exact simplex solvers based on rational arithmetic require a near-optimal warm start to be practical on large problems (current ME models have 70,000 constraints and variables and will grow larger). We have developed a quadruple-precision version of our linear and nonlinear optimizer MINOS, and a solution procedure (DQQ) involving Double and Quad MINOS that achieves reliability and efficiency for ME models and other challenging problems tested here. DQQ will enable extensive use of large linear and nonlinear models in systems biology and other applications involving multiscale data.

Constraint-Based Reconstruction and Analysis (COBRA)^1^ has been applied successfully to predict phenotypes for a range of genome-scale biochemical processes. The popularity of COBRA is partly due to the efficiency of the underlying optimization algorithms, permitting genome-scale modeling at a particular timescale using readily available open source software^2^,^3^ and industrial quality optimization algorithms^4^,^5^,^6^. A widespread application of COBRA is the modeling of steady states in genome-scale Metabolic models (M models). COBRA has also been used to model steady states in macromolecular Expression networks (E models), which stoichiometrically represent the transcription, translation, post-translational modification and formation of all protein complexes required for macromolecular biosynthesis and metabolic reaction catalysis^7^,^8^. COBRA of metabolic networks or expression networks depends on numerical optimization algorithms to compute solutions to certain model equations, or to determine that no solution exists. Our purpose is to discuss available options and to demonstrate an approach that is reliable and efficient for ever larger networks.

Metabolism and macromolecular Expression (ME) models have opened a whole new vista for predictive mechanistic modeling of cellular processes, but their size and multiscale nature pose a challenge to standard linear optimization (LO) solvers based on 16-digit double-precision floating-point arithmetic. Standard LO solvers usually apply scaling techniques^9^,^10^ to problems that are not already well scaled. The scaled problem typically solves more efficiently and accurately, but the solver must then unscale the solution, and this may generate significant primal or dual infeasibilities in the original problem (the constraints or optimality conditions may not be accurately satisfied).

A *lifting* approach^11^ has been implemented to alleviate this difficulty with multiscale problems. Lifting reduces the largest matrix entries by introducing auxiliary constraints and variables. This approach has permitted standard (double-precision) LO solvers to find more accurate solutions, even though the final objective value is still not satisfactory. Another approach to increasing the precision is to use an exact solver^12^. An exact simplex solver QSopt_ex^13^,^14^ has been used for a ME model of *Thermotoga maritima*^15^ (model TMA_ME) representing a network with about 18,000 metabolites and reactions. The solution time was about two weeks, compared to a few minutes for a standard double-precision solver, but the latter’s final objective value had only one correct digit. QSopt_ex has since been applied to a collection of 98 metabolic models by Chindelvitch *et al*.^16^ via their MONGOOSE toolbox. Most of the 98 models have less than 1000 metabolites and reactions. QSopt_ex required about a day to solve all models^16^, compared to a few seconds in total for a standard solver.

To advance COBRA for increasingly large biochemical networks, solvers that perform more efficiently than exact solvers and also perform more reliably than standard LO solvers are definitely needed. Gleixner *et al*.^17^,^18^,^19^,^20^ have addressed this need, and Chapter 4 of ref. 19 is devoted to multiscale metabolic networks, showing significant improvement relative to CPLEX^5^. Our work is complementary and confirms the value of enhancing the simplex solver in refs 17, 18, 19, 20 to employ quadruple-precision computation, as we have done here.

We use Single, Double, and Quad to denote the main options for floating-point arithmetic (with precision around 7, 16, and 34 digits respectively). For many years, scientific computation has advanced in two complementary ways: improved *algorithms* and improved *hardware*. Compilers have typically evaluated expressions using the same arithmetic as the variables’ data type. Most scientific codes apply Double variables and Double arithmetic throughout (16 significant digits stored in 64-bit words). The floating-point hardware often has *slightly extended precision* (80-bit registers). Kahan^21^ notes that early C compilers generated Double instructions for all floating-point computation *even for program variables stored in single precision*. Thus for a brief period, C programs were serendipitously more reliable than typical Fortran programs of the time. (For Single variables *a* and *b*, Fortran compilers would use Single arithmetic to evaluate the basic expressions *a* ± b, a*b, a/b, whereas C compilers would transfer *a* and *b* to longer registers and operate on them using Double arithmetic.) Most often, the C compiler’s extra precision was not needed, but occasionally it did make a critical difference. Kahan calls this the *humane* approach to debugging complex numerical software. Unfortunately, Quad hardware remains very rare and for the foreseeable future will be simulated on most machines by much slower software. Nevertheless, we believe the time has come to produce Quad versions of key sparse-matrix packages and large-scale optimization solvers for multiscale problems.

Here, we report the development and biological application of Quad MINOS, a quadruple-precision version of our general-purpose, industrial-strength linear and nonlinear optimization solver MINOS^22^,^23^. We also developed a Double-Quad-Quad MINOS procedure (DQQ) that combines the use of Double and Quad solvers in order to achieve a balance between efficiency in computation and accuracy of the solution. We extensively tested this DQQ procedure on 83 genome-scale metabolic network models (M models) obtained from the UCSD Systems Biology repository^24^,^25^ and 78 from the BiGG database^26^. We also applied DQQ to ME models of *Thermotoga maritima*^15^ (about 18,000 metabolites and reactions) and *E. coli* K12 MG1655^27^ (about 70,000 metabolites and reactions). For M models, we find that Double MINOS alone is sufficient to obtain non-zero steady-state solutions that satisfy feasiblility and optimality conditions with a tolerance of 10^−7^. For ME models, application of our DQQ procedure resulted in non-zero steady-state solutions that satisfy feasibility and optimality conditions with a tolerance of 10^−20^. The largest ME model required 4.5 hours, mostly in step D of DQQ because of conservative runtime options. Qsopt_ex would not be practical on such a large model unless warm-started at a near-optimal solution. The SoPlex80 bit solver^28^,^29^ has performed very efficiently on large ME models with the help of rational arithmetic at a near-optimal solution, but had difficulty on some other challenging problems that DQQ solved accurately (see ref. 19, Ch. 4], *problematic* models below, and Supplementary Information).

Thus, we expect our DQQ procedure to be a robust and efficient tool for the increasingly detailed study of biological processes, such as metabolism and macromolecular synthesis, and for challenging optimization problems arising in other scientific fields.

## Overview

A preliminary version of this work appeared in Ma and Saunders^30^. Here we name the approach DQQ and report experiments with an analogous but cheaper DRR procedure based on conventional iterative refinement of all linear equations arising in the simplex method (see Methods section and Supplementary Information). We also became aware of the work of Gleixner *et al*.^17^,^18^,^19^,^20^ and their thorough and successful implementation of iterative refinement in SoPlex80 bit. However, we learned that DRR may lose ground during periodic refactorizations of the simplex basis matrix *B*, if the current *B* is nearly singular and “basis repair” becomes necessary. Our DQQ and DRR experience points to the need for an optional Quad version of the basic SoPlex solver to ensure maximum reliability of the refinement approach in refs 17, 18, 19, 20. Meanwhile, DQQ will be effective on a wide range of problems as long as step D finishes naturally or is limited to a reasonable number of iterations before steps Q1 and Q2 take over.

## Results

We discuss Double and Quad implementations of MINOS applied to *linear optimization* (LO) problems of the form


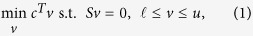


where *S* ∈ *R*^*m*×*n*^. To achieve reliability and efficiency on multiscale problems, we developed the following 3-step procedure.


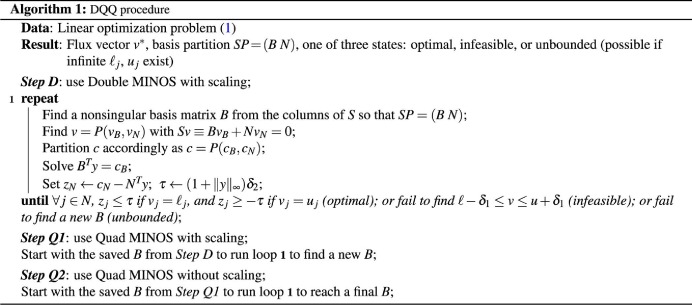


### DQQ procedure

*Step D* Apply the Double solver with scaling and somewhat strict runtime options.

*Step Q1* Warm-start the Quad solver with scaling and stricter options.

*Step Q2* Warm-start the Quad solver with no scaling but stricter options.

DQQ is described further in Algorithm 1, where loop **1** is the primal simplex method, *P* is a permutation matrix, and *δ*_1_, *δ*_2_ are Feasibility and Optimality tolerances. MINOS terminates loop **1** when the (possibly scaled) bounds on *v* are satisfied to within *δ*_1_, and the sign of 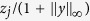 is correct to within *δ*_2_. Table 1 shows the default runtime options for Double MINOS and preferred options for each step of DQQ. Scale specifies whether the problem data should be scaled before the problem is solved (and unscaled afterward). Tolerances *δ*_1_, *δ*_2_ specify how well the primal and dual constraints of the (possibly scaled) problem should be satisfied. Expand frequency controls the MINOS anti-degeneracy procedure^31^. The LU tolerances balance stability and sparsity when LU factors of *B* are computed and updated.

Steps D and Q1 are usually sufficient, but Q2 costs little more and ensures that the tolerances *δ*_1_ and *δ*_2_ apply to the original (unscaled) problem. For conventional solvers it is reasonable to set *δ*_1_ and *δ*_2_ to 10^−6^ or perhaps as small as 10^−9^. For Quad MINOS, we set them to 10^−15^ to be sure of capturing variables *v*_*j*_ as small as *O*(10^−10^).

#### Small M models

Of the 98 metabolic network models in the UCSD Systems Biology repository^24^, A. Ebrahim was able to parse 83 models^32^ and compute solutions with a range of solvers^33^. We constructed MPS files for the 83 models^25^ and solved them via DQQ. Most models have less than 1000 metabolites and reactions. Almost all models solved in less than 0.08 seconds, and many in less than 0.01 seconds. The total time was less than 3 seconds. In contrast, ref. 16 reports that the exact solver Qsopt_ex needed a day.

#### Large ME models

COBRA can be used to stoichiometrically couple metabolic and macromolecular expression networks with single nucleotide resolution at genome-scale^15^,^27^. The corresponding Metabolic and macromolecular Expression models (ME models) explicitly represent catalysis by macromolecules, and in turn, metabolites are substrates in macromolecular synthesis reactions. These reconstructions lead to the first multi-timescale and genome-scale stoichiometric models, as they account for multiple cellular functions operating on widely different timescales and typically account for about 40 percent of a prokaryote’s open reading frames. A typical M model might be represented by 1000 reactions generated by hand^34^. In contrast, ME models can have more than 50,000 reactions, most of which have been generated algorithmically from template reactions (defined in the literature) and omics data^15^,^27^. Typical net metabolic reaction rates are 6 orders of magnitude faster than macromolecular synthesis reaction rates (millimole/gDW vs nanomole/gDW, gDW = gram dry weight), and the number of metabolic moieties in a macromolecule can be many orders of magnitude larger than in a typical metabolite. The combined effect is that the corresponding ME models have biochemically significant digits over many orders of magnitude. When Flux Balance Analysis (FBA) is augmented with coupling constraints^35^ that constrain the ratio between catalytic usage of a molecule and synthesis of the same molecule, the corresponding linear optimization problem is multiscale in the sense that both data values and solution values have greatly varying magnitudes. For a typical ME model, input data values (objective, stoichiometric or coupling coefficients, or bounds) differ by 6 orders of magnitude, and biochemically meaningful solution values can be as large as 10^8^ or as small as 10^−10^.

The results of DQQ on three large ME models are shown in Tables 2 and 3, including the model dimensions *m* and *n*, the number of nonzeros in *S*, the norms of the optimal primal and dual variables (v^*^, y^*^), the iterations and runtime for each step, the final objective value, and the primal and dual infeasibilities (Pinf and Dinf). The constraints in (1) are satisfied to within Pinf, and 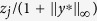 has the correct sign to within Dinf, where *B*^*T*^*y* = *c*_*B*_ for the optimal basis *B*, and *z* = *c* − *S*^*T*^*y*.

***TMA_ME*** developed by Lerman *et al*.^15^ has some large entries |*S*_*ij*_| and many small solution values *v*_*j*_ that have meanings to systems biologists. For example, transcription and translation rates can have values *O*(10^−10^) or less, which is much smaller than metabolic reactions. These small values are linked to large matrix entries arising from building large macromolecules from smaller constituents^27^. The ME part of the model also contains small |*S*_*ij*_|. For instance, enzyme levels are estimated in ME models by dividing certain metabolic fluxes by “effective rate constants.” Because these constants are typically large (e.g., 234,000 h^−1^), the matrix entries (the inverse of the rate constants) become small. In step D, most iterations were needed to find a feasible solution, with the objective then having the correct order of magnitude (but only one correct digit). Step Q1 improved the accuracy, and step Q2 provided confirmation. Note that the efficiency advantage of our approach is also evident: 385 seconds solve time for DQQ (Total time in Table 2) compared to 2 weeks using exact arithmetic^15^.

Two slightly different versions of this model provided welcome empirical evidence that the optimal objective and solution values do not change significantly when the problem data are perturbed by *O*(10^−6^) (see Supplementary Information).

***GlcAerWT*** is a ME model from the study by Thiele *et al*.^27^ After 33,000 iterations in step D, MINOS began to report singularities following updates to the basis factors (71 times during the next 15,000 iterations). After 47,718 iterations (D itns in Table 2), step D terminated with maximum primal and dual infeasibilities *O*(10^−4^) and *O*(1) (Pinf and Dinf in Table 3). These were small enough to be classified “Optimal”, but we see that the final objective value −6.7687e + 05 had no correct digits compared to −7.0382e + 05 in steps Q1 and Q2. For large models, step Q1 is important. It required significant work: 4,287 iterations costing 1958.9 seconds (Q1 itns and time in Table 2). Step Q2 soon confirmed the final objective value. The total time (12,599 seconds ≈ 3.5 hours) is modest compared to an expected time of months for the exact solver approach of ref. 16.

***GlcAlift*** was generated because of difficulties that TMA_ME and GlcAerWT presented to Double solvers. The lifting technique of ref. 11 was applied to GlcAerWT to reduce some of the large matrix values. The aim of lifting is to remove the need for scaling (and hence magnified errors from unscaling), but with DQQ we do activate scaling in step D because steps Q1 and Q2 follow. Our experience is that lifting improves accuracy for Double solvers but substantially increases the simplex iterations. On GlcAlift, Double MINOS again reported frequent singularities following basis updates (235 times starting near iteration 40,000). It took 93,857 iterations (D itns in Table 2), twice as many as GlcAerWT, with only a slight improvement in max{Pinf, Dinf} (Table 3). Double MINOS with scaling on the lifted model couldn’t reach agreement with the final objective −7.0434008750e + 05 in steps Q1 and Q2, and the total solve time increased (4.5 hours), mostly in step D. The objective for both GlcA models is to maximize *v*_60069_. The fact that there are no correct digits in the step D objectives illustrates the challenge that these models present, but steps Q1 and Q2 are accurate and efficient. The Q2 objectives for GlcAerWt and GlcAlift should be the same, but limited precision in the data files could explain why there is just 3-digit agreement.

The Tomlab interface^36^ and CPLEX were used by Thiele *et al*.^27^ to improve the results for standard Double solvers. On the NEOS server^37^, Gurobi was unable to solve GlcAerWT with default parameters (numeric error after nearly 600,000 iterations). It performed considerably better on GlcAlift (about 46,000 iterations) but terminated with a warning of unscaled primal/dual residuals 1.07 and 1.22e − 06. As shown above, our DQQ procedure saves researchers’ effort on lifting the model, and is able to solve the original model faster (3.5 hours vs 4.5 hours).

Further tests of the DQQ procedure on challenging LO problems are reported in **Methods**. As for the ME models, the simplex method in Double MINOS usually gives a good starting point for the same simplex method in Quad MINOS. Hence, much of the work can be performed efficiently with conventional 16-digit floating-point hardware to obtain near-optimal solutions. For Quad MINOS, 34-digit floating-point operations are implemented in the compiler’s Quad math library via software (on today’s machines). Each simplex iteration is therefore considerably slower than with floating-point hardware, but the reward is high accuracy. Of interest is that Quad MINOS usually achieves *much more accurate solutions than requested* (see bold figures in Table 3). This is a favorable empirical finding.

## Discussion

Exact solvers compute exact solutions to LO problems involving rational data. Although stoichiometric coefficients for chemical reactions are in principle integers, most genome-scale metabolic models have non-integer coefficients where the stoichiometry is known to only a few digits, e.g., a coefficient in a biomass reaction. Such a stoichiometric coefficient should not be considered exact data (to be converted into a rational number for use with an exact solver). This casts doubt on any effort to compute an exact solution for a particular FBA problem.

Exact solvers employ rational arithmetic, and have been applied to important problems^13^,^14^,^15^,^17^,^18^,^19^,^20^,^38^. Quad precision and variable-precision floating-point have also been mentioned^13^,^38^. Here, we exploit Quad precision more fully on a range of larger problems, knowing that current genome-scale models will continue to grow even larger.

While today’s commercial solvers (including CPLEX, Gurobi, Mosek, and Xpress^4^,^5^,^6^,^39^) are effective on a wide range of linear and mixed integer optimization models, the work of Thiele *et al*.^27^ calls for greater reliability in solving FBA and ME models in systems biology. Our DQQ procedure has demonstrated that warm starts with Quad solvers are efficient, and that the accuracy achieved exceeds requirements by a very safe margin. Kahan^21^ has noted that “*carrying somewhat more precision in the arithmetic than twice the precision carried in the data and available for the result will vastly reduce embarrassment due to roundoff-induced anomalies*” and that “*default evaluation in Quad is the humane option*,” as opposed to coding specialized tests for each application. The real(16) datatypes in today’s Fortran compilers provide a humane method for converting existing Double code to Quad. The float128 datatype in some C++ compilers makes it possible to switch from Double to Quad at runtime within a single code, making code maintenance even more humane.

Warm starts are essential for steps Q1 and Q2 of DQQ. Exact simplex solvers can also be warm-started, as noted by Gleixner *et al*.^18^,^19^. We could envisage a DE procedure: Double solver followed by Exact solver. However, for the GlcA problems in Table 2 (and for the gen problems in the Mészáros *problematic* set below), we see that step Q1 performs a significant number of iterations. Thus, warm-starting an exact solver on large models may not be practical when the Double solver is not reliable.

Looking ahead, we note that metabolic reconstructions of the form (1) may need to be processed before they can be treated as stoichiometrically consistent models. As discussed in ref. 40, certain rows of *S* may need to be deleted according to the solution 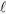 of the problem 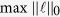 s.t. 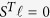, 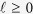. This problem can be approximated by the linear problem


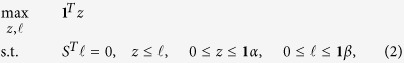


where scalars *α, β* are proportional to the smallest molecular mass considered non-zero and the largest molecular mass allowed (e.g., *α* = 10^−4^, *β* = 10^4^). Note that problem (2) involves *S*^*T*^ and is larger than the FBA problem (1) itself. We could not design consistent FBA models in this way unless we were sure of being able to solve (2) effectively. Our work here offers assurance of such capability.

We believe that reliable solutions are now readily available for large, multiscale applications such as FBA and flux variability analysis (FVA) in systems biology^1^,^27^,^35^,^41^,^42^, and that our DQQ procedure will allow biologists to build increasingly large models to explore metabolism and macromolecular synthesis. Combined use of Double and Quad solvers will help other areas of computational science involving multiscale optimization problems. For example, Dattorro^43^ describes an approach to analog filter design that requires a Quad optimization solver to deal with a wide range of frequencies that must be raised to high powers. Like ME models with nonlinear constraints (7), this application can be treated with Quad precision and binary search on a sequence of problems. We have also treated nonlinear constraints directly with the nonlinear algorithms in Quad MINOS^23^,^44^.

## Methods

### Multiscale constraint-based modeling

Consider a network of biochemical reactions, represented by a stoichiometric matrix 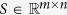 with each row and column corresponding to a molecular species and biochemical reaction, respectively. *S*_*ij*_ respresents the *stoichiometry* of molecular species *i* participating as a substrate (negative) or product (positive) in reaction *j*. The evolution of molecular species concentrations with respect to time (*t*) is given by the ordinary differential equation


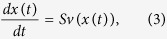


where 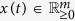 is a vector of time-dependent concentrations and 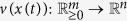 is a nonlinear function of concentrations that depends on the kinetic mechanism of each reaction.

If one assumes that species concentrations are time-invariant, then the set of all steady-state reaction rates, satisfying *Sv*(*x*) = 0, may be approximated by the linear *steady-state constraint Sv* = 0, where 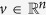 is a vector of reaction fluxes. Thermodynamic principles and experimental data can also be used to specify lower and upper *bound constraints* on reaction fluxes 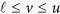. Biochemical relationships between the rates of macromolecular synthesis and utilization can be approximated by coupling of the corresponding reaction fluxes^35^, e.g., pyruvate kinase reaction flux and the synthesis flux of pyruvate kinase in a ME model^27^. Flux coupling can be represented by bounding the ratio between two reaction fluxes with two coupling coefficients:


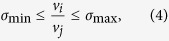


where *v*_*i*_ and *v*_*j*_ are a pair of non-negative fluxes. This nonlinear constraint can be reformulated into a pair of linear *coupling constraints*


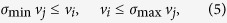


or more generally a set of linear inequalities *Cv* ≤ *d*. In addition to the aforementioned physicochemical and biochemical contraints, one may hypothesize a biologically motivated objective. For example, in modeling a growing cell, one may hypothesize that the objective is to maximize the rate of a biomass synthesis reaction. Typically, a biomass synthesis reaction is created with experimentally determined stoichiometric coefficients, each of which represents the relative composition of a cellular biomass constituent. Optimization of a linear combination of reaction fluxes *c*^*T*^*v* leads to linear optimization problems: (1). Flux balance analysis of a ME model with coupling constraints results in an ill-scaled instance of this problem because the stoichiometric coefficients and coupling coefficients vary over many orders of magnitude.

### MINOS implementation

MINOS^22^,^23^ is a linear and nonlinear optimization solver implemented in Fortran 77 to solve problems of the form


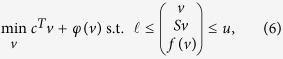


where *ϕ*(*v*) is a smooth nonlinear function and *f*(*v*) is a vector of smooth nonlinear functions (see Supplementary Information).

### Further tests of DQQ

We report results from the primal simplex solvers in Double and Quad MINOS on two sets of challenging LO problems shown in Table 4. As with the M and ME models, we used an Apple iMac with 2.93 GHz quad-core Intel i7 and gfortran compiler with -O flag (GNU Fortran 5.2.0). The input files were in the MPS format of commercial mathematical programming systems^45^ with 12-character fields for data values.

#### The pilot problems

These are economic models developed by Professor George Dantzig in the Systems Optimization Laboratory at Stanford University during the 1980s. They have been used in other computational studies (e.g. ref. 38) and are available from Netlib^46^. We use three examples of increasing size: pilot4, pilot, pilot87. In Table 5, three lines for each problem show the results of steps D, Q1, Q2 of the DQQ procedure.

For pilot, line 1 shows that step D (cold start and scaling) required 16060 iterations and 9 CPU seconds. The unscaled solution *v* satisfied the constraints in (1) to within *O*(10^−6^) and the dual solution *y* satisfied the optimality conditions to within *O*(10^−3^). Line 2 shows that step Q1 needed only 29 further Quad iterations and 0.3 seconds to obtain a very accurate solution. Line 3 shows that the “insurance” step Q2 with no scaling gave an equally good solution (with maximum infeasibilities 0.0 and *O*(10^−32^)). The final Double and Quad objective values differ in the 4th significant digit, as suggested by the *O*(10^−3^) dual infeasibility in step D.

For pilot4 and pilot87 the results are analogous.

#### The Mészáros problematic problems

Our DQQ procedure was initially developed for this set of LO problems collected by Mészáros^47^, who named them *problematic* and noted that “*modeling mistakes made these problems “crazy,” but they are excellent examples to test numerical robustness of a solver*.” The first two problems have large entries in *S*. The step D objective value for de063155 has only 1 digit of precision, and none for de063157. Nevertheless, the infeasibilities Pinf and Dinf for steps Q1 and Q2 are small when the solution norms are taken into account.

The gen problems arise from image reconstruction. There are no large entries in *S, v, y*, but the primal solutions *v* are highly degenerate. For gen1, 60% of the step D and Q1 iterations made no improvement to the objective, and 30% of the basic variables in the final solution are on their lower bound. Step Q1 gave an almost feasible initial solution (253 basic variables outside their bounds by more than 10^−15^ with a sum of infeasibilities of *O*(10^−8^)), yet over 200,000 iterations were needed to reach optimality. Evidently Quad precision does not remove the need for a more rigorous anti-degeneracy procedure (such as Wolfe’s method as advocated by Fletcher^48^) or steepest-edge pricing^49^ to reduce the total number of iterations. Problems gen1 and gen4 show that step Q2 is sometimes needed to achieve high accuracy.

Problem l30 behaved similarly (80% degenerate iterations in steps D and Q1). Since the objective value is essentially zero, we can’t expect the Q1 and Q2 objectives to agree. The Q1 iterations were inadvertently limited to 500,000, but step Q2 did not have much further to go.

Problem iprob is artificial and intended to be feasible with a very ill-conditioned optimal basis, but the MPS file contained low-precision data such as 0.604 or 0.0422. The Double and Quad runs determine that the problem is infeasible. This is an example of Quad removing doubt that would inevitably arise with just Double.

Table 5 shows that Quad MINOS usually achieves much greater accuracy than requested (the primal and dual infeasibilities are almost always much smaller than 10^−15^). Thus our procedure for handling the *problematic* problems has seemed appropriate for the systems biology M and ME models. Like the gen problems, the ME models showed many degenerate iterations in step D, but fortunately not so many total iterations in step Q1 (see Table 2). This is important for FVA and for ME models with nonlinear constraints, which involve multiple warm starts.

#### ME models (FBA with coupling constraints)

In these models, coupling constraints are often functions of the organism’s growth rate *μ*. Thus, O’Brien *et al*.^50^ consider growth-rate optimization nonlinearly, with *μ* entering as the objective in (1) instead of via a linear biomass objective function. Nonlinear constraints of the form


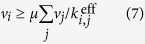


are added to (1), where *v*_*i*_, *v*_*j*_, *μ* are all variables, and 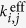 is an effective rate constant. If *μ* is fixed at a specific value *μ*_*k*_, the constraints (7) become linear. O’Brien *et al*.^50^ implemented a binary search on a discrete set of values within an interval 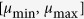 to find the largest *μ*_*k*_ ≡ μ^*^ that keeps the associated linear problem feasible. The procedure required reliable solution of a sequence of LO problems.

#### Flux Variability Analysis (FVA)

After FBA (1) returns an optimal objective value *c*^*T*^v^*^ = *Z*_0_, FVA examines how much a flux *v*_*j*_ can vary within the feasible region without much change to the optimal objective:


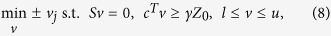


where 0 < *γ* < 1 and *γ* ≈ 1. Potentially 2*n* LO problems (8) must be solved if all reactions are of interest. Warm starts are used when *j* is increased to *j* + 1^42^. For such a sequence of problems it would be simplest to warm-start each problem in Quad, but warm-starting in Double and then Quad might be more efficient.

### Conventional iterative refinement

A Double simplex solver would be more reliable with the help of iterative refinement (Wilkinson^51^) on each linear system involving the basis matrix *B* or its transpose, but we found this inadequate for the biology models (see DRR procedure in Supplementary Information).

### The zoom strategy

A step toward warm-starting interior methods for optimization was proposed in ref. 52 to take advantage of the fact that a low-accuracy solution (*x*_1_, *y*_1_) for a general problem


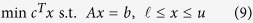


can be obtained relatively cheaply when an iterative solver for linear systems is used to compute each search direction. (The iterative solver must work harder as the interior method approaches a solution.) If (*x*_1_, *y*_1_) has at least some correct digits, the primal residual *r*_1_ = *b* − *Ax*_1_ will be somewhat small (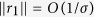 for some *σ* ≫ 1) and the dual residual *d*_1_ = *c* − *A*^*T*^*y*_1_ will be comparably small in the elements associated with the final *B*. If we define


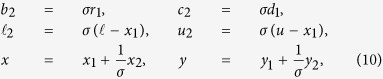


and note that the problem is equivalent to


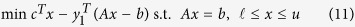


with dual variable *y* − *y*_1_, we see that *x*_2_ solves


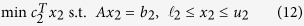


with dual variable *y*_2_. Importantly, with *σ* chosen carefully we expect (*x*_2_, *y*_2_) in this “*zoomed in*” problem to be of order 1. Hence we can solve the problem with the same solver as before (as solvers use absolute tolerances and assume that *A* and the solution are of order 1). If the computed (*x*_2_, *y*_2_) has at least some digits of accuracy, the correction 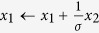, 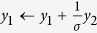 will be more accurate than before. The process can be repeated. With repeated zooms (named *refinement rounds* in refs 18 and 19), the residuals (*r*_1_, *d*_1_) must be computed with increasingly high precision. Subject to the expense of using rational arithmetic for this purpose, ref. 18 gives extensive results for over 1000 challenging problems and shows that exceptional accuracy can be obtained in reasonable time: only 3 or 4 refinements to achieve 10^−50^ precision, and less than 20 refinements to achieve 10^−250^. SoPlex80 bit^28^,^29^ is used for each refinement round with feasibility and optimality tolerances set to 10^−9^. In ref. 18 the authors recognize that much depends on the robustness of the simplex solver used for the original problem and each refinement. The potential difficulties are the same as in each step of our DRR procedure, where Double MINOS is on the brink of failure on the Glc problems because *B* is frequently near-singular when it is refactorized every 100 iterations. A practical answer for ref. 18 is to use a more accurate floating-point solver such as Quad MINOS (or Quad versions of SoPlex or SNOPT^53^) for all refinement rounds.

### DQQ serves the current purpose

In the context of ME models whose non-integer data is accurate to only 4 or 5 digits, we don’t need 10^−50^ precision. Tables 3 and 5 show that our DQQ procedure achieves more accuracy than necessary on all tested examples. For models where the Double solver is expected to encounter difficulty, step D can use a reasonable iteration limit. Step Q1 will perform more of the total work with greatly improved reliability. Step Q2 provides a small but important improvement at negligible cost, ensuring small residuals for the original (unscaled) problem.

### The need for Quad precision

To summarize why a conventional Double solver may not be adequate for multiscale problems (even with iterative refinement on systems *Bp* = *a* and *B*^*T*^*y* = *c*_*B*_ each iteration), we note that the current basis matrix *B* must be factorized at regular intervals. If *B* appears to be nearly singular, a “basis repair” procedure replaces some columns of *B* by appropriate unit vectors (thus making certain slack variables basic). The new *B* is better conditioned, but the solution obtained after recomputing the basic variables from *Bv*_*B*_ + *Nv*_*N*_ = 0 may have an objective value *c*^*T*^*v* that is unpredictably less optimal than before. The preceding iterations would make progress, but basis repair allows loss of ground. Basis repair is unlikely to happen if Quad precision is used for all storage and computation, as it is in steps Q1 and Q2 of DQQ.

### Data and software availability

Double and Quad Fortran 77 implementations of MINOS are included within the Cobra toolbox^2^. MPS or JSON files for all models discussed are available from ref. 25. Python code for running Double and Quad MINOS on the BiGG JSON files is also available from ref. 25.

## Additional Information

**How to cite this article**: Ma, D. *et al*. Reliable and efficient solution of genome-scale models of Metabolism and macromolecular Expression. *Sci. Rep.* **7**, 40863; doi: 10.1038/srep40863 (2017).

**Publisher's note:** Springer Nature remains neutral with regard to jurisdictional claims in published maps and institutional affiliations.

## Supplementary Material

Supplementary Information

## Figures and Tables

**Table 1:** Runtime options for MINOS in each step of the DQQ procedure.

	Default	Step D	Step Q1	Step Q2
Precision	Double	Double	Quad	Quad
Scale	Yes	Yes	Yes	No
Feasibility tol *δ*_1_	1e − 6	1e − 7	1e − 15	1e − 15
Optimality tol *δ*_2_	1e − 6	1e − 7	1e − 15	1e − 15
Expand frequency	10000	100000	100000	100000
LU Factor tol	100.0	1.9	10.0	5.0
LU Update tol	10.0	1.9	10.0	5.0

**Table 2:** Three large ME biochemical network models TMA_ME, GlcAerWT, GlcAlift^11^,^15^,^27^.

ME model	TMA_ME	GlcAerWT	GlcAlift
*m*	18210	68300	69529
*n*	17535	76664	77893
nnz(*S*)	336302	926357	928815
max |*S*_*ij*_|	2.1e + 04	8.0e + 05	2.6e + 05
||v*||_∞_	5.9e + 00	6.3e + 07	6.3e + 07
||y*||_∞_	1.1e + 00	2.4e + 07	2.4e + 07
D itns	21026	47718	93857
D time	350.9	10567.8	15913.7
Q1 itns	597	4287	1631
Q1 time	29.0	1958.9	277.3
Q2 itns	0	4	1
Q2 time	5.4	72.1	44.0
Total time	385	12599	16235

Dimensions of *m* × *n* constraint matrices *S*, size of the largest optimal primal and dual variables v*, y^*^, number of iterations and runtimes in seconds for each step, and the total runtime of each model.

**Table 3:** Three large ME biochemical network models TMA_ME, GlcAerWT, GlcAlift^11^,^15^,^27^.

ME model	Step	Objective	Pinf	Dinf
TMA_ME	D	8.3789966820e − 07	−06	−05
	Q1	8.7036315385e − 07	−25	−32
	Q2	8.7036315385e − 07	**—**	−**32**
GlcAerWT	D	−6.7687059922e + 05	−04	+00
	Q1	−7.0382449681e + 05	−07	−26
	Q2	−7.0382449681e + 05	−**21**	−**22**
GlcAlift	D	−5.3319574961e + 05	−03	−01
	Q1	−7.0434008750e + 05	−08	−22
	Q2	−7.0434008750e + 05	−**18**	−**23**

Optimal objective value of each step, Pinf and Dinf = final maximum primal and dual infeasibilities (log_10_ values tabulated, except – means 0). Bold figures show the final *(step Q2)* Pinf and Dinf.

**Table 4:** Three pilot models from Netlib^46^ and eight *problematic* problems from Mészáros^47^.

model	*m*	*n*	nnz(*S*)	max|*S*_*ij*_|	||v*||_∞_	||y*||_∞_
pilot4	411	1000	5145	2.8e + 04	9.6e + 04	2.7e + 02
pilot	1442	3652	43220	1.5e + 02	4.1e + 03	2.0e + 02
pilot87	2031	4883	73804	1.0e + 03	2.4e + 04	1.1e + 01
de063155	853	1488	5405	8.3e + 11	3.1e + 13	6.2e + 04
de063157	937	1488	5551	2.3e + 18	2.3e + 17	6.2e + 04
de080285	937	1488	5471	9.7e + 02	1.1e + 02	2.6e + 01
gen1	770	2560	64621	1.0e + 00	3.0e + 00	1.0e + 00
gen2	1122	3264	84095	1.0e + 00	3.3e + 00	1.0e + 00
gen4	1538	4297	110174	1.0e + 00	3.0e + 00	1.0e + 00
l30	2702	15380	64790	1.8e + 00	1.0e + 09	4.2e + 00
iprob	3002	3001	12000	9.9e + 03	3.1e + 02	1.1e + 00

Dimensions of *m* × *n* constraint matrices *S*, size of the largest nonzero in *S*, and norm of the optimal primal and dual variables v^*^, y^*^.

**Table 5:** Iterations and runtimes in seconds for steps D, Q1, Q2 on the problems of Table 4.

model	Itns	Times	Final objective	Pinf	Dinf
pilot4	1464	0.1	−2.5811392619e + 03	−05	−12
	7	0.0	−2.5811392589e + 03	−52	−31
	0	0.0	−2.5811392589e + 03	—	−**29**
pilot	16060	9.0	−5.5739887685e + 02	−06	−03
	29	0.3	−5.5748972928e + 02	—	−32
	0	0.1	−5.5748972928e + 02	**—**	−**32**
pilot87	19340	22.6	3.0171038489e + 02	−08	−06
	32	0.9	3.0171034733e + 02	—	−32
	0	0.6	3.0171034733e + 02	**—**	−**33**
de063155	973	0.1	1.8968895791e + 10	−14	+03
	90	0.1	9.8830944565e + 09	—	−27
	0	0.0	9.8830944565e + 09	**—**	−**24**
de063157	1473	0.1	2.6170359397e + 12	—	+08
	286	0.2	2.1528501109e + 07	−29	−12
	0	0.0	2.1528501109e + 07	**—**	−**12**
de080285	418	0.0	1.4495817688e + 01	−09	−02
	132	0.1	1.3924732864e + 01	−35	−32
	0	0.0	1.3924732864e + 01	**—**	−**32**
gen1	303212	156.9	−8.1861282705e − 08	−06	−13
	216746	3431.2	1.2939275026e − 06	−12	−31
	8304	112.5	1.2953925804e − 06	−**46**	−**31**
gen2	45905	60.0	3.2927907833e + 00	−04	−12
	2192	359.9	3.2927907840e + 00	—	−29
	0	10.4	3.2927907840e + 00	**—**	−**32**
gen4	38111	151.3	−1.2724113149e − 07	−07	−12
	58118	6420.2	2.8932557999e − 06	−12	−31
	50	4.3	2.8933064888e − 06	−**53**	−**30**
l30	1302602	805.6	9.5266141670e − 01	−08	−09
	500000	6168.8	−4.5793509329e − 26	−25	−00
	16292	204.4	−6.6656750251e − 26	−**25**	−**31**
iprob	1087	0.2	2.6891551285e + 03	+02	−11
	0	0.0	2.6891551285e + 03	+02	−30
	0	0.0	2.6891551285e + 03	+02	−**28**

Pinf and Dinf = final maximum primal and dual infeasibilities (log_10_ values tabulated, except **–** means 0). Problem iprob is infeasible. Bold figures show Pinf and Dinf at the end of step Q2. Note that Pinf/||v*||_∞_ and Dinf/||y*||_∞_ are *O*(10^−30^) or smaller, even though only *O*(10^−15^) was requested.
