# Supplementary material for: Reliable and efficient solution of genome-scale models of Metabolism and macromolecular Expression
Source: Sci Rep. 2017 Jan 18;7:40863. doi: 10.1038/srep40863 (PMC5241643; doi:10.1038/srep40863)
Supplement: Supplementary Information [file srep40863-s1.pdf]

Reliable and efficient solution of genome-scale models of Metabolism and macromolecular Expression

Ding Ma[1], Laurence Yang[2], Ronan M. T. Fleming[3], Ines Thiele[3], Bernhard O. Palsson[2,4], and Michael A. Saunders[1,*]

[1]Stanford University, Dept of Management Science and Engineering, Stanford, CA 94305, USA
[2]University of California, San Diego, Dept of Bioengineering, La Jolla, CA 92093, USA
[3]University of Luxembourg, Luxembourg Centre for Systems Biomedicine, L-4365 Esch-sur-Alzette, Luxembourg
[4]Technical University of Denmark, Novo Nordisk Foundation Center for Biosustainability, 2970 Hørsholm, Denmark
[*]saunders@stanford.edu (Scientific Reports (SREP-16-20266A), qLP30, December 10, 2016)

ABSTRACT

This is Supplementary Information for the above-named article.

Introduction

Figure 1 summarizes our DQQ procedure for achieving reliability and efficiency for multiscale optimization problems.

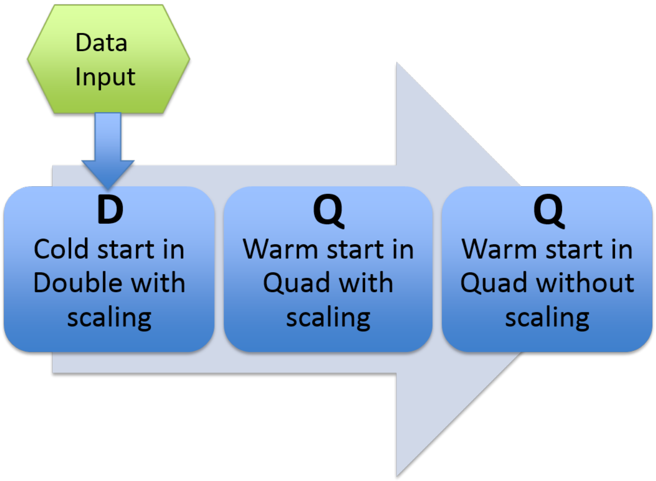

Figure 1. Flowchart for the 3-step DQQ procedure.

The main paper reports application of DQQ to three large ME models (TMA_ME, GlcAerWT, GlcAlift) and to some other challenging linear optimization problems (the pilot economic models and the Mészáros *problematic* set). Below we provide the following supplementary information:

- Solution of 78 Metabolic models by Double and Quad MINOS, verifying that the Double solver gives reliable results.
- Solution of two slightly different forms of the TMA_ME model, showing robustness of solution values with respect to $O(10^{-6})$ relative perturbations of the data.
- Some details of the Double and Quad MINOS implementations.
- Experiments with conventional iterative refinement (DRR procedure).
- Results with Gurobi on the ME models.
- Results with SoPlex80bit on the ME and *problematic* models.

Table 1. TMA_ME model. Robustness of objective values computed by four high-accuracy solvers for two slightly different versions of the problem with 13-digit and 6-digit data (from Matlab and MPS data respectively).

	Optimal objective	
SoPlex80bit	8.703671403e-07	Matlab data
QSopt_ex	8.703646169e-07	Matlab data
Quad SQOPT	8.703646169e-07	Matlab data
Quad MINOS	8.703631539e-07	MPS data

Table 2. TMA_ME model. Robustness of small solution values v_j and w_j computed by Quad MINOS for two slightly different versions (Matlab and MPS data respectively).

j	107	201	302
v_j	2.336815e-06	8.703646e-07	1.454536e-11
w_j	2.336823e-06	8.703632e-07	1.454540e-11

Metabolic models with Quad solvers admit biomass synthesis

COBRA models of metabolic networks assume the existence of at least one steady-state flux vector that satisfies the imposed constraints and admits a non-zero optimal objective. Where the objective is to maximize a biomass synthesis reaction, the corresponding FBA problem should admit a nonzero biomass synthesis rate. It is established practice to solve monoscale metabolic FBA problems with Double solvers, so one may ask: do biomass synthesis predictions from metabolic models hold when higher precision solvers are applied to the same FBA problem? We tested 78 M models derived from the BiGG database[1] using Double and Quad MINOS. We downloaded these models in the JSON format and parsed them using the JSON reader in cobrapy[2]. The models were not modified after loading, so all constraints, bounds, and objective coefficients were used as in the original files. All models were feasible using both Double and Quad, and all but five models had an optimal objective value greater than zero. Of these five models, four simply had all-zero objective coefficients, while the remaining (RECON1) model maximized a single reaction (S6T14g) but its optimal value was zero. The maximum difference in objective value between Double and Quad was 2.6×10^{-12}. The additional precision provided by Quad MINOS enabled us to conclude efficiently and effectively that the 78 metabolic models could be solved reliably using a Double solver. This conclusion is consistent with previous findings by Ebrahim et al.[3].

Robustness of solution values for TMA_ME

TMA_ME[4] was the first ME model that we used for Quad experiments. The data S, c, ℓ, u came as a Matlab structure with $c_j = 0$, $\ell_j = 0$, $u_j = 1000$ for most j, except four variables had smaller upper bounds, the last variable had moderate positive bounds, and 64 variables were fixed at zero. The objective was to maximize flux v_{17533}. We output the data to a plain text file. Most entries of S were integers (represented exactly), but about 5000 S_{ij} values were of the form 8.037943687315e-01 or 3.488862338191e-06 with 13 significant digits. The text data was read into Double and Quad versions of a prototype Fortran 90 implementation of SQOPT[5].

For the present work, we used the same Matlab data to generate an MPS file for input into MINOS. Since this is limited to 6 significant digits, the values in the preceding paragraph were rounded to 8.03794e-01 and 3.48886e-06 and in total about 5000 S_{ij} values had $O(10^{-6})$ relative perturbations of this kind. This was a fortuitous limitation for the ME models. We have been concerned that such data perturbations could alter the FBA solution greatly because the final basis matrices could have condition number as large as 10^6 or even 10^{12} (as estimated by LUSOL[6] each time SQOPT or MINOS factorizes the current basis B). However, in comparing Quad SQOPT and Quad MINOS with SoPlex[7,8] and the exact simplex solver QSopt_ex[9], we observe in Table 1 that the final objective values for TMA_ME in Matlab data reported by QSopt_ex and Quad SQOPT match in every digit. Moreover, the objective value achieved by Quad MINOS on the perturbed data in MPS format agrees to 5 digits of the results from the exact solver QSopt_ex on the "accurate" data. These results show the robustness of the TMA_ME model and our 34-digit Quad solvers.

More importantly, for the most part *even small solution values* are perturbed in only the 5th or 6th significant digit. Let v and w be the solutions obtained on slightly different data. Some example values are given in Table 2. Among all j for which $\max(v_j, w_j) > \delta_1 = 10^{-15}$ (the feasibility tolerance), the largest relative difference $|v_j - w_j| / \max(v_j, w_j)$ was less than 10^{-5} for all but 31 variables. For 22 of these pairs, either v_j or w_j was primal or dual degenerate (meaning one of them was zero and there are alternative solutions with the same objective value). The remaining 9 variables had v_j, w_j values shown in Table 3.

We see that the values are small (the same magnitude as the data perturbation) but for each of the nine pairs there is about 1

Table 3. TMA_ME model. The values of 9 fluxes v_j, w_j computed by Quad MINOS for two slightly different versions of the problem, revealing robustness of all 9 solution pairs. These values have 1 digit of agreement. Almost all 17535 pairs of values agree to 5 or more digits.

j	v_j	w_j	Relative difference
16383	6.07e-07	2.04e-06	0.70
16459	1.71e-06	2.18e-06	0.22
16483	2.47e-06	5.99e-07	0.76
16730	1.44e-06	7.87e-07	0.46
17461	1.71e-06	2.18e-06	0.22
17462	2.47e-06	5.99e-07	0.76
17478	6.07e-07	2.04e-06	0.70
17507	1.44e-06	7.87e-07	0.46
17517	8.70e-07	2.97e-06	0.71

digit of agreement. We could expect thousands of small solution pairs to differ more, yet for almost *all* 17535 pairs at least 5 digits agree.

Although these observations do not prove robustness of FBA models in general (because we analyzed only one perturbation to one model), they are welcome empirical evidence that the solutions are not extremely unstable. Quad solvers can help evaluate the robustness of future (increasingly large) models of metabolic networks by enabling similar comparison of high-accuracy solutions for slightly different problems.

MINOS implementation

MINOS[10,11] is a linear and nonlinear optimization solver implemented in Fortran 77 to solve problems of the form

$$\min_{v} \; c^T v + \varphi(v) \;\; \text{s.t.} \;\; \ell \le \begin{pmatrix} v \\ Sv \\ f(v) \end{pmatrix} \le u, \tag{1}$$

where $\varphi(v)$ is a smooth nonlinear function and $f(v)$ is a vector of smooth nonlinear functions. The matrix S and the Jacobian of $f(v)$ are assumed to be sparse.

Let Single/Double/Quad denote the floating-point formats defined in the 2008 IEEE 754 standard[12] with about 7/16/34 digits of precision, respectively. Single is not useful in the present context, and Double may not ensure adequate accuracy for multiscale problems. This is the reason for our work. Since release 4.6 of the GCC C and Fortran compilers[13], Quad has been available via the `long double` and `real(16)` data types. Thus, we have made a Quad version of Double MINOS using the GNU gfortran compiler (GNU Fortran 5.2.0).

On today's machines, Double is implemented in hardware, while Quad (if available) is typically implemented in a software library, in this case GCC libquadmath[14].

For Double MINOS, floating-point variables are declared `real(8)` ($\approx$ 16 digits). For Quad MINOS, they are `real(16)` ($\approx$ 34 digits) with the data S, c, ℓ, u stored in Quad even though they are not known to that precision. This allows operations such as Sv and $S^T y$ to be carried out directly on the elements of S and the Quad vectors v, y. If S were stored in Double, such products would require each entry S_{ij} to be converted from Double to Quad at runtime (many times).

The primal simplex solver in MINOS includes geometric mean scaling[15], the EXPAND anti-degeneracy procedure[16], and partial pricing (but no steepest-edge pricing, which would generally reduce total iterations and time). Basis LU factorizations and updates are handled by LUSOL[6]. Cold starts use a Crash procedure to find a triangular initial basis matrix. Basis files are used to preserve solutions between runs and to enable warm starts.

Scaling is commonly applied to linear programs to make the scaled data and solution values closer to 1. Feasibility and optimality tolerances can be chosen more easily for the scaled problem, and LU factors of the basis matrix are more likely to be sparse. For geometric mean scaling, several passes are made through the columns and rows of S to compute a scale factor for each column and row. A difficulty is that the scaled problem may solve to within specified feasibility and optimality tolerances, but when the solution is unscaled it may lie significantly outside the original (unscaled) bounds.

EXPAND tries to accommodate consecutive "degenerate" simplex iterations that make no improvement to the objective function. The problem bounds are effectively expanded a tiny amount each iteration to permit nonzero improvement. Convergence is usually achieved but is not theoretically guaranteed[17]. Progress sometimes stalls for long sequences of iterations.

LUSOL bounds the subdiagonals of L when the current basis matrix B is factorized as $P_1 B P_2 = LU$ with some permutations P_1, P_2. It also bounds off-diagonal elements of elementary triangular factors L_j that update L in product form each simplex

Table 4. DRR procedure on three ME models. Iterations and runtimes in seconds for step D (Double MINOS with scaling) and steps R1, R2 (Double MINOS with iterative refinement, with and without scaling). Pinf and Dinf = final maximum primal and dual infeasibilities ($\log_{10}$ values tabulated). Bold figures show Pinf and Dinf at the end of step R2. The fourth line for each model shows the correct objective value (from step Q2 of DQQ).

model	Itns	Times	Final objective	Pinf	Dinf
TMA_ME	21026	350.9	8.3789966820e-07	−06	−05
	422	25.4	8.6990918717e-07	−08	−07
	71	0.0	8.7035701805e-07	**−10**	**−10**
			8.7036315385e-07		
GlcAerWT	47718	10567.8	−6.7687059922e+05	−04	+00
	907	1442.7	−7.0344344753e+05	−04	−04
	157	151.2	−7.0344342883e+05	**−10**	**−02**
			−7.0382449681e+05		
GlcAlift	19340	15913.7	−5.3319574961e+05	−03	−01
	447	198.8	−7.0331052509e+05	−03	−03
	460	0.6	−7.0330602383e+05	**−06**	**−10**
			−7.0434008750e+05		

iteration. (The diagonals of L and each L_j are implicitly 1.) Maximum numerical stability would be achieved by setting the LU Factor and Update tolerances to be near 1.0, but larger values are typically chosen to balance stability with sparsity. For safety, we specify 1.9 in step D of DQQ. This value guards against unstable factorization of the deceptive matrix $\mathrm{diag}(-1\ 2\ 1)$, and improves the reliability of Double MINOS in the present context.

Conventional iterative refinement

For the biology models, our aim is to satisfy Feasibility and Optimality tolerances of 10^{-15} (close to Double precision). It is reasonable to suppose that this could be achieved within a Double simplex solver by implementing iterative refinement (Wilkinson[18]) for every linear system involving the basis matrix B or B^T. This is a more sparing use of Quad precision than our DQQ procedure. For example, each time the current B is factorized directly (typically a new sparse LU factorization every 100 iterations), the constraints $Sv = 0$ can be satisfied more accurately by computing the primal residual $r = 0 - Sv$ from the current solution v, solving $B\Delta v_B = r$, and updating $v_B \leftarrow v_B + \Delta v_B$. In general, the new v will not be significantly more accurate unless r is computed in Quad. (If B is nearly singular, more than one refinement may be needed.) Similarly for solving $B^T y = c_B$ after refactorization, and for two systems of the form $Bp = a$ and $B^T y = c_B$ each iteration of the simplex method.

By analogy with DQQ, we implemented the following procedure within a test version of Double MINOS. Note that "iterative refinement" in steps R1, R2 means a single refinement for each B or B^T system, with residuals $-Sv$, $a - Bp$, $c_B - B^T y$ computed in Quad as just described.

DRR procedure

Step D Apply Double MINOS with scaling and moderately strict runtime options.
Step R1 Warm-start Double MINOS with scaling, stricter options, and iterative refinement.
Step R2 Warm-start Double MINOS without scaling but with stricter options and iterative refinement.

Step D is the same as for DQQ (with no refinement). The runtime options for each step are the same as for DQQ, except in steps R1, R2 the tolerances 1e-15 were relaxed to 1e-9.

In Table 4 we see that this simplified (cheap) form of iterative refinement is only partially successful, with step R2 achieving only 4, 3, and 2 correct digits in the final objective. For GlcAerWT, steps R1 and R2 encountered frequent near-singularities in the LU factors of B (requiring excessive refactorizations and alteration of B), and in step R2, the single refinement could not always achieve full Double precision accuracy for each system. Additional refinements would improve the final Pinf and Dinf, but would not reduce the excessive factorizations. We conclude that on the bigger ME problems, a Double solver is on the brink of failure even with the aid of conventional (Wilkinson-type) iterative refinement of each system involving B and B^T. We conclude that our DQQ procedure is a more expensive but vitally more robust approach.

Results with NEOS/Gurobi

For large linear models, commercial solvers have reached a high peak of efficiency. It would be ideal to make use of them to the extent possible. For example, their Presolve capability allows most of the optimization to be performed on a greatly reduced form of any typical model.

Table 5. Performance of Gurobi with default options on three ME models. Note that "switch to quad" means switch to 80-bit floating-point (not to IEEE Quad precision). This did not help GLcAerWT. For GlcAlift2, the options were NumericFocus 3, no Presolve, and no scaling.

TMA_ME	Presolve	$18209 \times 17535 \to 2386 \times 2925$
Optimal	Iterations	1703
0.5 secs	Objective	9.6318438361e-07
	True obj	8.7036315385e-07
GlcAerWT	Presolve	$68299 \times 76664 \to 18065 \times 26157$
	Warning	switch to quad (itns ≈ 14000)
Numeric error	Iterations	593819
3715 secs	Objective	3.2926249e+07
	True obj	-7.0382449681e+05
GlcAlift	Presolve	$69528 \times 77893 \to 18063 \times 26155$
	Warning	switch to quad (itns ≈ 10000)
Optimal	Iterations	45947
109 secs	Objective	-7.043390954e+05
	True obj	-7.0434008750e+05
	Warning	unscaled primal/dual residuals: 1.07, 1.22e-06
GlcAlift2		
Optimal	Iterations	128596
844 secs	Objective	-7.043415774e+05
	True obj	-7.0434008750e+05
	Warning	unscaled primal residual: 1.05e-05

Table 6. Performance of SoPlex80bit on three large ME models (default options except no simplifier or lifting).

TMA_ME		18209×17535
Optimal	Iterations	19563 (3 refinements)
90.9 secs	Objective	8.7036315385e-07
	True obj	8.7036315385e-07
GlcAerWT		68299×76664
Optimal	Iterations	86366 (3 refinements)
1059 secs	Objective	-7.0382449681e+05
	True obj	-7.0382449681e+05
GlcAlift		69528×77893
Optimal	Iterations	83941 (3 refinements)
889 secs	Objective	-7.0434008750e+05
	True obj	-7.0434008750e+05

Table 5 summarizes the performance of Gurobi[19] on three large ME models via the NEOS server[20]. The first three results used Gurobi's default runtime options, including Presolve, Dual simplex, and Scaling (with default FeasibilityTol = OptimalityTol = 1e-6). TMA_ME seemed to solve successfully, but from the Quad MINOS solution we know that Gurobi's final objective value has no correct digits. GlcAerWT failed with "Numeric error" after many expensive iterations using 80-bit floating-point. GlcAlift also switched to 80-bit floating-point. The scaled problem seemed to solve successfully, but unscaling damaged the primal residual and this casts significant doubt on the final solution. (This is the reason for our research.)

For GlcAlift2 we specified NumericFocus 3 with no Presolve and no scaling. These options are appropriate for lifted models[21]. Gurobi did not switch to 80-bit arithmetic, yet achieved 5 correct digits in the objective. This helps confirm the value of the lifting strategy of[21], and would provide a good starting point for steps Q1 and Q2 of DQQ. However, DQQ permits us to solve the original model GlcAerWT directly (without the lifting transformation).

Results with NEOS/SoPlex80bit

Table 6 summarizes the performance of SoPlex80bit[8] on the three large ME models via NEOS with default options, except the simplifier and lifting options were turned off to ensure that SoPlex80bit was iterating on the same problems as MINOS.

SoPlex80bit performed extremely well on all ME models (Table 6). The first floating-point solves achieved maximum primal and dual feasibilities of order 1e-7 or less, with no sign of scaling or potentially troublesome unscaling, and three rounds of iterative refinement reduced the infeasibilities to order 1e-44(!). The optimal objective values agreed to the 11 digits printed by Quad MINOS. Analogous excellent performance by SoPlex80bit on large ME models is described by Gleixner [22, Ch. 4].

Table 7. Performance of SoPlex80bit on the *problematic* models (default options except no simplifier or lifting).

de063155		852 × 1488
Optimal	Iterations	1766 (3 refinements)
0.3 secs	Objective	`9.8830944565e+09`
	True obj	`9.8830944565e+09`
de063157		936 × 1488
Optimal	Iterations	3828 before refinement
0.1 secs	Objective	`2.15277062e+07`
	True obj	`2.1528501109e+07`
de080285		936 × 1488
	Iterations	804 (2 refinements)
0.1 secs	Objective	`1.3924732864e+01`
	True obj	`1.3924732864e+01`
gen1		769 × 2560
	Iterations	12850 (3 refinements)
186.5 secs	Objective	`1.2953925804e-06`
	True obj	`1.2953925804e-06`
gen2		1121 × 3264
Optimal	Iterations	12079 (2 refinements)
6016 secs	Objective	`3.2927907840e+00`
	True obj	`3.2927907840e+00`
gen4		1537 × 4297
Optimal	Iterations	14358 (3 refinements)
7132 secs	Objective	`2.8933064888e-06`
	True obj	`2.8933064888e-06`
l30		2701 × 15380
	Iterations	3400093 before refinement
11552 secs	Objective	`-2.54658516e-11`
	True obj	`-6.6.......e-26`
iprob		3001 × 3001
Infeasible	Iterations	3001 (2 refinements)
0.6 secs	Objective	`1.0e+100`

On the *problematic* set (Table 7), SoPlex80bit solved most problems solved accurately, but with some anomalies. On de063157, the first floating-point solve achieved 5 significant digits in the objective function but with primal and dual infeasibilities of 4e+2 and 1e+4. The first refinement reduced the latter to 2e+1 and 3e-12, and the second refinement achieved 4e-15 and 3e-12. This should have been acceptable, but a further 100 refinements were conducted (at negligible cost) before the run was terminated with no final solution available.

On gen2 and gen4, the first floating-point solves were very efficient and accurate (41 and 82 seconds respectively). Three refinements achieved primal and dual infeasibilities of order 1e-11 or less. A final rational factorization proved expensive and accounted for 99% of the total times (6016 and 7132 seconds respectively), but confirmed optimality.

On l30, the first floating-point solve performed many iterations but achieved primal and dual infeasibilities of 4e-9 and 1e-10 with objective value −2.5e-11, which should have been acceptable. The first refinement reported numerical troubles after 2702 iterations. It continued to about 154000 iterations and computed an unbounded ray. Nearly 4000 refinements followed (each doing no iterations) before numerical trouble was reported. One final solve performed 3000 iterations before increasing the Markowitz threshold and terminating with no solution.

Details of this nature will change, but some of them hint at the need for higher precision in the floating-point solver to facilitate SoPlex's iterative refinement.

Looking ahead

The large-scale optimizer SNOPT[5] is maintained as a Fortran 77 solver `snopt7`[23] suitable for step D of the DQQ procedure. An accompanying Fortran 2003 version `snopt9` has also been developed, for which Double and Quad libraries can be built with only one line of source code changed. They are ideal for applying DQQ to future multiscale linear and nonlinear optimization models, as long as step D can be terminated early enough when numerical difficulties arise. Quad enhancements to the SoPlex floating-point solver also promise reliability and extreme accuracy for future challenging models.

References

1. King, Z. A. *et al.* BiGG Models: A platform for integrating, standardizing, and sharing genome-scale models. *Nucl. Acids Res.* **44**, D515–522 (2016).
2. Ebrahim, A., Lerman, J. A., Palsson, B. O. & Hyduke, D. R. COBRApy: COnstraints-Based Reconstruction and Analysis for Python. *BMC Syst Biol* **7**, 74 (2013).
3. Ebrahim, A. *et al.* Do genome-scale models need exact solvers or clearer standards? *Mol Syst Biol* **11**, 831 (2015).
4. Lerman, J. A. *et al.* In silico method for modelling metabolism and gene product expression at genome scale. *Nat. Commun.* **3**, 10 pp. (2012).
5. Gill, P. E., Murray, W. & Saunders, M. A. SNOPT: An SQP algorithm for large-scale constrained optimization. *SIAM Review* **47**, 99–131 (2005). SIGEST article.
6. LUSOL sparse LU factorization package (2013). URL http://stanford.edu/group/SOL/software/lusol. (Date of access: 27/04/2014).
7. Wunderling, R. *Paralleler und objektorientierter Simplex-Algorithmus*. Ph.D. thesis, Technische Universität Berlin (1996).
8. Soplex: The sequential object-oriented simplex solver (2016). URL http://soplex.zib.de. (Date of access: 16/06/2016).
9. Applegate, D., Cook, W., Dash, S. & Mevnkamp, M. QSopt_ex: A simplex solver for computing exact rational solutions to LP problems (2008). URL http://www.math.uwaterloo.ca/~bico/qsopt/ex. (Date of access: 13/10/2016).
10. Murtagh, B. A. & Saunders, M. A. Large-scale linearly constrained optimization. *Math. Program.* **14**, 41–72 (1978).
11. Murtagh, B. A. & Saunders, M. A. A projected Lagrangian algorithm and its implementation for sparse nonlinear constraints. *Math. Program. Study* **16**, 84–117 (1982).
12. IEEE Std 754-2008. IEEE Computer Society (2008).
13. GCC, the GNU Compiler Collection (2016). URL https://gcc.gnu.org/. (Date of access: 27/04/2016).
14. The GCC Quad-precision math library application programming interface (API) (2016). URL http://gcc.gnu.org/onlinedocs/libquadmath/. (Date of access: 27/04/2016).
15. Fourer, R. Solving staircase linear programs by the simplex method, 1: Inversion. *Math. Program.* **23**, 274–313 (1982).
16. Gill, P. E., Murray, W., Saunders, M. A. & Wright, M. H. A practical anti-cycling procedure for linear and nonlinear programming. *Math. Program.* **45**, 437–474 (1989).
17. Hall, J. A. J. & McKinnon, K. I. M. The simplest examples where the simplex method cycles and conditions where EXPAND fails to prevent cycling. *Math. Progam., Ser. B* **100**, 133–150 (2004).
18. Wilkinson, J. H. *The Algebraic Eigenvalue Problem* (Oxford University Press, 1965).
19. Gurobi optimization system for linear and integer programming (2014). URL http://www.gurobi.com. (Date of access: 01/02/2016).
20. NEOS server for optimization (2016). URL http://www.neos-server.org/neos/. (Date of access: 02/01/2016).
21. Sun, Y., Fleming, R. M. T., Thiele, I. & Saunders, M. A. Robust flux balance analysis of multiscale biochemical reaction networks. *BMC Bioinformatics* **14** (2013).
22. Gleixner, A. M. *Exact and Fast Algorithms for Mixed-Integer Nonlinear Programming*. Ph.D. thesis, Konrad-Zuse-Zentrum für Informationstechnik Berlin (ZIB), Technical University of Berlin (2015).
23. UCSD/Stanford optimization software (2016). URL http://ccom.ucsd.edu/~optimizers/. (Date of access: 03/05/2016).
